# Light-Dependent Resistors as Dosimetric Sensors in Radiotherapy

**DOI:** 10.3390/s20061568

**Published:** 2020-03-11

**Authors:** Juan Román-Raya, Isidoro Ruiz-García, Pablo Escobedo, Alberto J. Palma, Damián Guirado, Miguel A. Carvajal

**Affiliations:** 1 Instituto de Investigación Biosanitaria, Ibs.Granada. Hospital Universitario Clínico San Cecilio, 18160 Granada, Spain; juan.roman.raya.sspa@juntadeandalucia.es (J.R.-R.);; 2 ECsens, Department of Electronics and Computer Technology, Sport and Health University Research Institute (iMUDS), University of Granada, 18071 Granada, Spain; isirg@correo.ugr.es (I.R.-G.); ajpalma@ugr.es (A.J.P.); 3 Bendable Electronics and Sensing Technologies (BEST) Group, Electronics and Nanoscale Engineering, University of Glasgow, Glasgow G128QQ, UK; pablo.escobedo@glasgow.ac.uk; 4 CIBER de Epidemiología y Salud Pública (CIBERESP), 18016 Granada, Spain

**Keywords:** dosimetry, radiotherapy, light-dependent resistor, dose rate, thermal characterization

## Abstract

Safe quality control of radiotherapy treatments lies in reliable dosimetric sensors. Currently, ionization chambers and solid-state diodes along with electrometers as readout systems are accomplishing this task. In this work, we present a well-known and low-cost semiconductor sensor, the light-dependent resistor (LDR), as an alternative to the existing sensing devices for dosimetry. To demonstrate this, a complete characterization of the response to radiation of commercial LDRs has been conducted in terms of sensitivity, reproducibility and thermal correction under different bias voltages. Irradiation sessions have been applied under the common conditions in radiotherapy treatments using a hospital linear accelerator. Moreover, the same electrometer used for the ionization chamber has also been successfully used for LDRs. In comparison with the sensitivity achieved for the ionization chamber (0.2 nC/cGy at 400 V bias voltage), higher sensitivities have been measured for the proposed LDRs, ranging from 0.24 to 1.04 nC/cGy at bias voltages from 30 to 150 V, with a reproducibility uncertainty among samples of around 10%. In addition, LDR temperature dependence has been properly modeled using the simple thermistor model so that an easy thermal drift correction of dose measurements can be applied. Therefore, experimental results show that LDRs can be a reliable alternative to dosimetric sensors with the advantages of low size, affordable cost and the fact that it could be adopted with minimal changes in routine dosimetry quality control since the same readout system is fully compatible.

## 1. Introduction

Radiotherapy is one of the most common techniques to treat solid cancers. Providing the prescribed doses is critical to ensure that tumor cells are killed while normal tissues are preserved. Currently, one of the most used dosimetry systems in radiotherapy is the ionization chamber due to its simplicity and reliability [1,2]. Its principle of operation is based on the collection of the ions produced by ionizing radiation into the chamber cavity, usually filled with air. To avoid the ion recombination, an electric field is applied and the collected charge produces a current which can be measured. The ionization chamber is connected to an electrometer both to create the necessary electric field by means of a bias voltage and to measure the charge induced by the radiation, integrating the current. Therefore, the electrometer is a type of source monitor unit (SMU) capable of biasing in a wide voltage range (up to several hundred volts) and measuring currents in the range of nanoamperes. 

Other devices also widely used in radiotherapy are the semiconductor-based dosimeters [2,3,4,5,6]. These devices take advantage of their small size, comparatively reduced cost and lower or no bias voltage requirements. The use of semiconductor electronic devices for in vivo dosimetry has a long tradition in the field of radiotherapy [7]. In most cases, devices specifically designed for dosimetry are usually manufactured using specialized procedures to provide them with high sensitivity to radiation. Such is the case of PIN diodes or radiation-sensitive field-effect-transistors (RADFETs). A RADFET is essentially a MOSFET transistor whose structure has been optimized to increase the sensitivity to radiation by means of a thicker gate oxide and manufactured with an oxidative process that makes it more sensitive [8,9]. The cost of these systems would be significantly reduced provided that commercial devices not specifically manufactured for dosimetry could be used, which has already been addressed for MOSFET transistors [10,11]. Recently, other semiconductor devices have been studied for the same purpose, such as photodiodes and phototransistors [12,13,14], and in some cases the record of absorbed doses has been achieved in radiodiagnosis tests [15]. Some authors have observed that variations of the reverse current arise in certain photodiodes and phototransistors subjected to a field of ionizing radiation, proving that such variations are proportional to the absorbed dose rate [15,16].

A light-dependent resistor (LDR) (also called a photoresistor or photoconductive cell) is a light-controlled variable resistor whose resistance depends on the incident light intensity. It is a semiconductor device manufactured from compounds of CdSe, CdS, InSb or PbS [17,18]. When photons with energy higher than the semiconductor band gap are absorbed by the material, electron–hole pairs are generated both by band-to-band transitions and by transitions involving forbidden gap energy levels (also named trap levels). The resulting free charge carriers conduct electricity, thereby reducing the resistance. An LDR is typically applied in light-sensitive detector circuits and light-switching circuits. It is widely known that LDRs reduce their resistance with the increasing light intensity following a non-linear trend close to a 1/R dependence. A similar non-linear behavior has been measured with dose rates under ionizing radiation as well [19,20]. In addition, if the radiation response of LDRs could be measured using a clinical electrometer such as those commonly used for reading ionizing chambers, the cost of the complete measurement system would be significantly reduced by removing the need for a specific reader system.

In this work, three models of commercial LDRs have been characterized as dosimetric sensors for radiation beams typically used in radiotherapy. Their responses have been measured using both digital multimeters (DMM) and clinical electrometers available in hospitals. From the measured generated current and its time integration, both the dose rate and the accumulated dose can be calculated. Our aim is to present and characterize low-cost LDR-based dosimeters with suitable performances. In this work we also show that these LDR-based dosimeters could be read by clinical electrometers without using any specific readout system, thus avoiding the additional costs that the introduction of novel sensors would entail.

## 2. Materials and Methods

**Figure 1:**
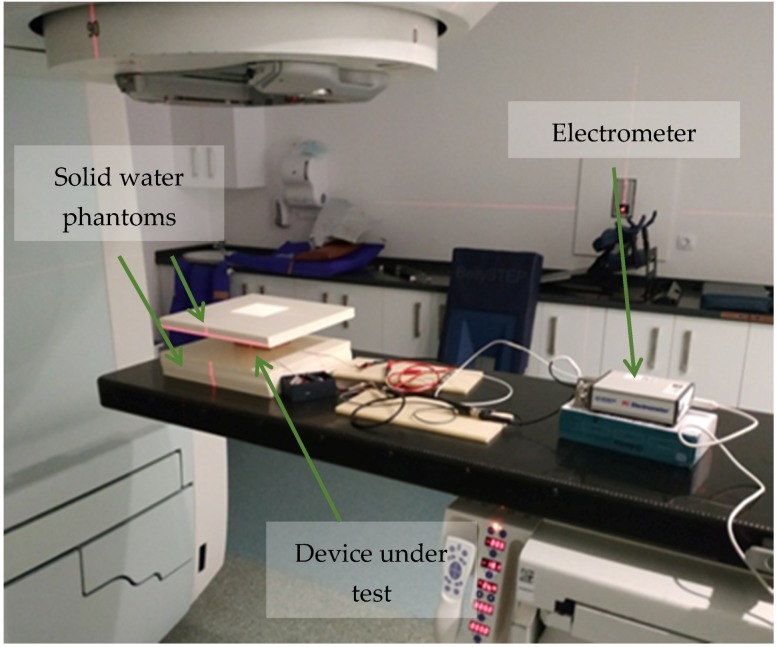
Experimental setup for the characterization of the LDRs radiation response. Solid water layers are shown under and over the box containing the device under test.

Three commercial light-dependent resistor models have been characterized as dosimetric sensors for radiation X-ray beams typically used in radiotherapy. Three samples of each model have been studied. The experimental characterization included the measurement of their response to radiation in terms of resistance variation and current/charge generated under bias voltage. Moreover, LDR temperature dependence has also been analyzed as the main interferent with the goal of achieving thermal compensation of the measured parameters. The selected LDRs were the NORPS-12 (Silonex Inc., UK), the NSL-19M51 (Luna Optoelectronics, USA) and the VT43N2 (Excelitas Technologies, USA), all of them made of CdS, encapsulated in a moisture-resistant coating and enclosed in a plastic casing. Farmer-type ionization chamber PTW 30010 (PTW, Freiburg, Germany), very common in radiotherapy dosimetry, with a volume of 0.6 cm^3^, was used as the reference dosimeter for benchmarking. Each device under test was painted with black nail polish and introduced into a box in order to shield it from environmental light. Irradiation sessions were held at the “Hospital Universitario Clínico San Cecilio” of Granada (Spain). The characterization process involved three different phases:

1. Preliminary study: NORPS-12 and NSL-19M51 models were irradiated in darkness with an 18 MV X-ray beam produced by a LINAC Mevatron KDS (Siemens AG, Germany) at dose rates of 50, 100, 150, 200, 250 and 300 cGy/min, placing a 10 × 10 cm field at the LINAC isocenter. To avoid backscattering effects, six centimeters of solid water were located underneath and three phantoms over the devices under test to reach the electronic equilibrium conditions. In this study, real-time resistance measurements were carried out with a benchtop digital multimeter (DMM34410, Agilent Technologies, Santa Clara, USA). The multimeter was placed out of the treatment room and the devices were connected using standard wiring without shielding.
2. Main study: As the readout system, the multimeter was replaced by a clinical electrometer (PC Electrometer, Sun Nuclear, USA) originally designed to operate with ionization chambers. The electrometer used is able to provide a bias voltage between –400 V and +400 V in steps of 1 V and has a measured current range up to 50 nA with a charge resolution of 15 fC. Irradiation sessions were carried out with a different linear accelerator, Artiste (Siemens AG, Germany). The LDRs were located at the isocenter of the radiation under solid water to reach electronic equilibrium. They were irradiated with a 6 MV X-ray beam and dose rates from 50 cGy/min to 300 cGy/min. The devices under test were also biased by the electrometer to study the effect of the bias voltage on their response to radiation. A TNC connector was employed to connect the LDRs to the electrometer and polarize the devices with voltages from 30 V to 150 V. The electrometer was placed inside the treatment room to reduce the noise produced by the wiring. It was controlled by a computer connected with a USB extension cable and placed out of the treatment room. With this experimental setup, the NSL-19M51 and VT43N2 LDR devices were tested. Five solid water phantoms with a thickness of 1 cm each were placed under the box containing the LDRs, two more solid water phantoms over it to reach the electronic equilibrium conditions for 6 MV, as it is shown in Figure 1.
3. Finally, the temperature dependence of the selected models VT43N2 and NSL-19M51 was assessed. To conduct the thermal characterization of the LDRs, current–voltage characteristic curves were accurately measured at different temperatures. In order to maintain minimum luminosity and infrared radiation, the photoresistors were introduced in a Faraday cage and covered with aluminum foil. The semiconductor analyzer B1500 (Agilent Technologies, Santa Clara, USA) was used to measure the I–V characteristics of the photoresistors. This instrument has a measurement range from 0.1 fA to 1 A, and 0.5 µV to 200 V. Temperature sweeps from 0 to 50 °C were conducted with a climate chamber VCL4006 (Vötsch Industrietechnik, Balingen-Frommern, Germany), capable of varying the temperature from −40 to 180 °C with a temperature deviation in time of ±0.3–1.0 K and a temperature homogeneity in space of ±0.5–2.0 K. To monitor the temperature more accurately, a digital thermometer RS 408-6109 (Amidata S.A., Madrid, Spain) with an accuracy of 0.1 °C was placed inside the chamber close to the LDRs.

## 3. Results and Discussion

### 3.1. Dosimetric Characterization with Digital Multimeter

**Figure 2:**
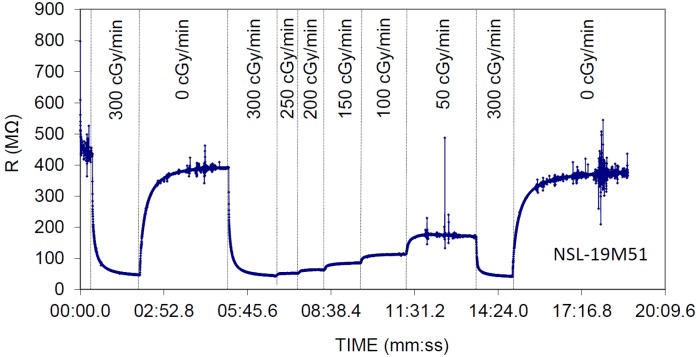
Experimental resistance of the NSL-19M51 LDR at different dose rates. Vertical lines limit the different dose rate time intervals during irradiation, from 300 cGy/min down to 50 cGy/min, including non-irradiation periods (0 cGy/min).

Figure 2 shows the typical transient resistance of the NSL-19M51 LDR under different dose rates. The delayed responses in the resistance values that can be observed in Figure 2 have two sources: The slow device response mainly in dark conditions [20]; and the acquisition and averaging period, which is automatically set by the multimeter for measuring high resistance values (>100 MΩ). Time constant can be well above several seconds. Moreover, as previously reported [20], a significant decrease in the resistance value from its initial level is observed in Figure 2 (notice the resistance decay mainly in the 0 cGy/min periods). This is due to a performance degradation of non-irradiated devices caused by radiation damages. This issue will be examined at the end of the next subsection.

**Figure 3:**
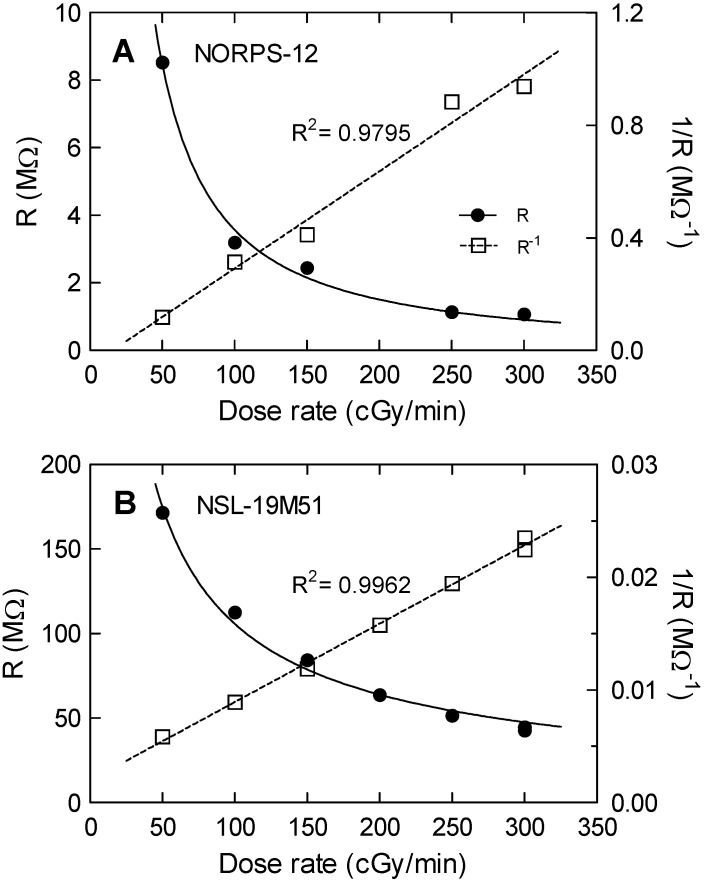
Experimental resistance (symbols) as a function of dose rate for the two models of LDRs under test: (**A**) NORPS-12 and (**B**) NSL-19M51. Solid and dashed trend lines are plotted for R and R^−1^ respectively.

Mean resistances as a function of the dose rate for both models are depicted in Figure 3. Mean resistances were calculated as the average values in the steady-state regions. The uncertainty was taken as the quadratic error propagation of the standard deviation at the flat zone (steady-state resistance) and the experimental uncertainty. As a result, a linear dependence of R^−1^ with dose rate was found for both models, as it was previously shown [20]. Therefore, both LDR devices can be considered as suitable dosimeters. However, the NORPS-12 presented lower linearity and a significant drift of the dark resistance (without irradiation). By contrast, the NSL-19M51 LDR showed no significant drift and a linear dependence of the R^−1^ with dose rate (R^2^ = 0.996) between 50 and 300 cGy/min. Therefore, the NORPS-12 was discarded for further characterization mainly due to its high dark resistance drift.

### 3.2. Dosimetric Characterization with a Clinical Electrometer

**Figure 4:**
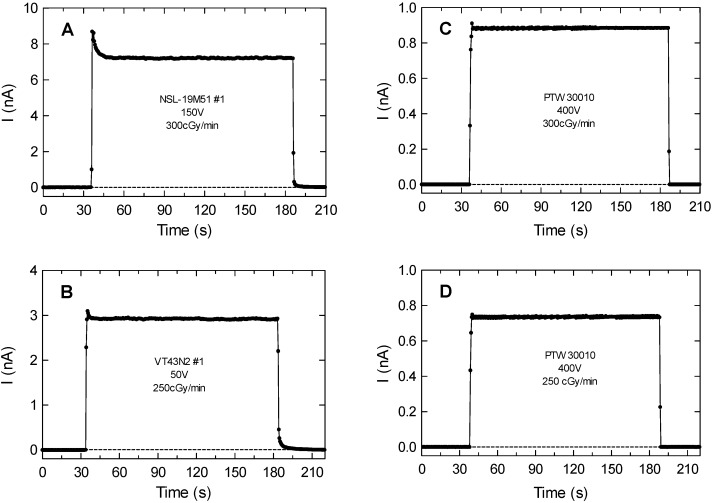
(**A**) Current transients versus time for NSL-19M51 number #1 biased at 150 V and dose rate of 300 cGy/min, and (**B**) VT43N2 number #1 biased at 50 V with dose rate of 250 cGy/min. (**C**) and (**D**) Current transients of ionization chamber PTW 30010 biased at 400 V with dose rates of 300 and 250 cG/min respectively. The horizontal line corresponds to three standard deviations of the base line current values. In all the cases the uncertainty corresponds to a coverage factor k = 2, which cannot be seen because they are smaller than the symbols used to represent the experimental data.

After the previous preliminary tests with the benchtop multimeter, the LDR devices NSL-19M51 and VT43N2 were biased and measured while irradiated using a clinical electrometer. Figure 4A,B, show two transients of the current measured for an NSL-19M51 sample biased at 150 V with a dose rate of 300 cGy/min and a VT43N2 sample biased at 50 V with a dose rate of 250 cGy/min, respectively. The responses to the radiation of the LDRs under test are similar to those of the ionization chamber depicted in Figure 4C,D. Differences shown at the beginning of the transitory are caused by capacitive effects of the LDR structure. As mentioned above, when incident X-rays fall on the LDR surface, carriers are generated either by band-to-band transitions or by transitions involving forbidden gap energy levels (also named trap levels), resulting in a conductivity increase [20]. To the best of our knowledge, these initial sharp peaks observed in Figure 4A,B have not been previously studied in this context. We have experimentally found that the initial amplitude of this peak increases with the polarization voltage and is different from one LDR device to another. We believe that these peaks are due to generated carriers from trap levels. The peak decays are showing the emptying of these traps. When they are fully discharged, the steady-state current generated by band-to-band transitions remain. The peak amplitude dependence on the polarization voltage can be explained by the more efficient electron/hole separation before recombination at higher voltages than for the steady-state induced current. 

The irradiation period is considered when the measured current is higher than three times the standard deviation of the base-line value (dashed lines in Figure 4). Considering the bias voltage and performing the sensor calibration, both the dose rate and the accumulated dose can be extracted from the current transients as those shown in Figure 4:

1. Dose rate can be calculated from the average steady-state current (ASC), defined as the current increment from the base-line without irradiation to the steady-state value in the flat region during irradiation. Uncertainties were estimated as the quadratic error propagation of twice the standard deviation of the measurements at the flat zone and the electrometer sensitivity.
2. Accumulated dose can be extracted from the charge produced by irradiation (CPI) calculated as the time integral of the current in the irradiation period defined above.

LDR sensitivity to radiation was defined as the ratio between the generated charge and absorbed dose in nC/cGy.

**Figure 5:**
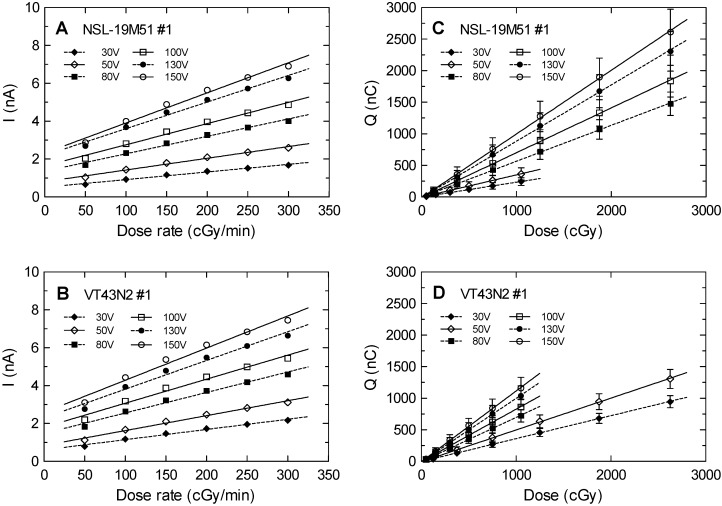
(**A**) and (**B**) Average steady-state current, ASC, versus dose rate; and (**C**) and (**D**) generated charge, CPI, versus accumulated dose for both models (NSL-19M51 number #1 and VT43N2 number #1) and with different bias voltages. Uncertainties correspond to a coverage factor k = 2, but the error bars are smaller than the dot symbols. Lines correspond to the linear tendencies for each bias voltage.

Figure 5 shows the calibration curves of the average steady-state current and the dose rate at different bias voltages for the two LDRs under study, the NSL-19M51 number #1 and VT43N2 number #1. Linear tendencies were obtained as expected from the preliminary study. At constant voltage, current and resistance are inversely proportional, and therefore the same trend was expected representing ASC or R^−1^ versus the dose rate (see Figure 3). Higher slopes with the bias voltage are also observed. This can be explained due to a lower photocarrier recombination at higher voltages (higher electric field), allowing higher free carrier concentration and therefore a higher output current.

**Figure 6:**
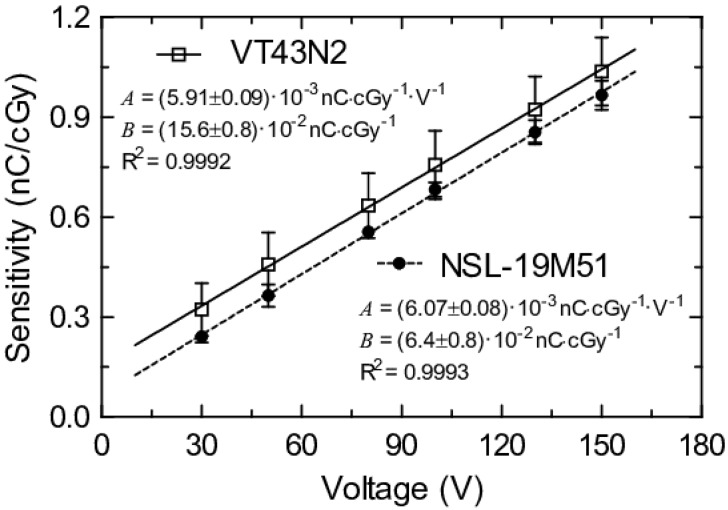
LDR sensitivity versus bias voltage for both studied models (NSL-19M51 number #1 and VT43N2 number #1). Experimental data are represented by symbols and linear trend lines (*y = A·x + B*) are also shown.

Sensitivities and correlation factors are summarized in Table 1 for dose rate and accumulated dose calculations. The minimum correlation factor (R^2^) was 0.986 (VT43N2 biased at 130 V); thus, it can be considered that the current produced is proportional to the dose rate in the analyzed range. Regarding the sensitivity, both LDR devices presented a reproducibility error around 10%. However, their sensing areas are different. The sensing area of the VT43N2 model is bigger than the sensing area of the NSL-19M51, therefore it can provide a higher current with the same bias voltage. If low size sensors are required, the NSL-19M51 would be more suitable. Figure 6 depicts the dependences of the sensitivity with the bias voltage showing excellent linear behavior. It is interesting to note that both slopes of this figure, A, present very similar values, which is consistent with their similar composition. On the other hand, the intercepts, B, may be different due to dissimilar dimensions and dark current issues, which are closely related. 

Regarding the sensor degradation observed in Figure 2, following the procedure that is conducted to reduce this effect on other radiation detectors such as diodes, a dose of 120 Gy was applied to one NSL-19M51 sample. This is usually named pre-irradiation session, and it is used to improve the detector reproducibility by deliberately increasing and making prevalent the radiation-induced trap concentration among samples, before its use as radiation sensor [21]. After this pre-irradiation session, the LDR showed a performance degradation lower than 1% for an accumulated dose of 10 Gy when biased at 150 V.

### 3.3. Thermal Compensation

**Figure 7:**
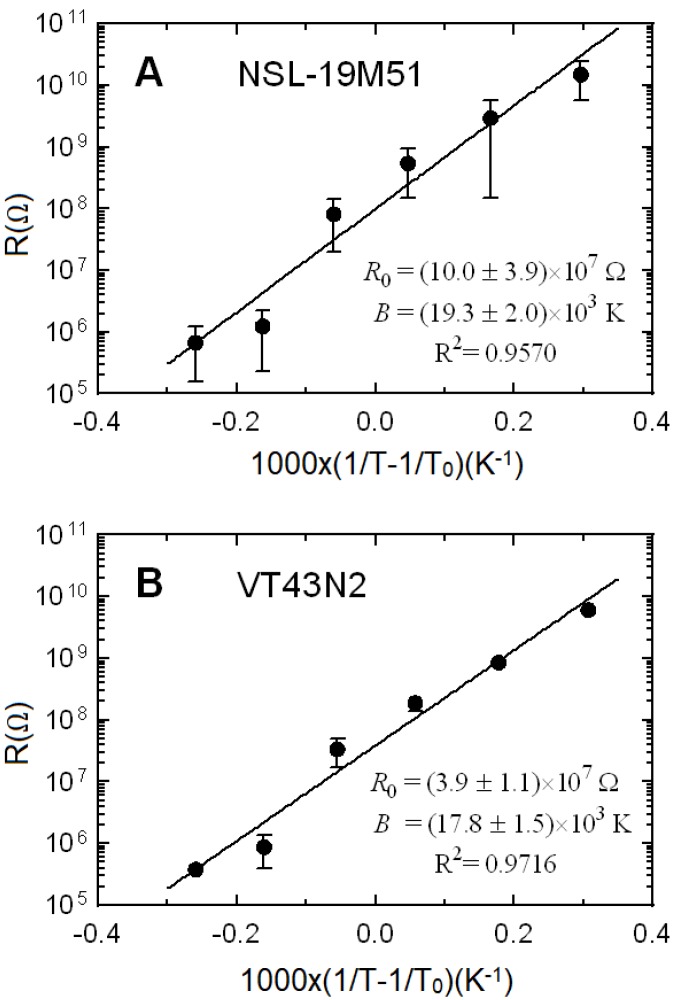
Average resistance of the three samples vs. the inverse of the temperature according to model of Equation (1) for (**A**) NSL-19M51 and (**B**) VT43N2 LDRs. Resistances at the reference temperature, *R*_0_, and characteristic temperature, *B*, are also shown. Uncertainties correspond to a coverage factor k = 2, and in some cases error bars cannot be seen because they are smaller than the symbol used to represent the experimental data.

A semiconductor material, cadmium sulfide in this case, is the main component of an LDR. In the temperature range analyzed in this work, its conductivity increases with temperature following a non-linear behavior [17,22]. Indeed, an exponential tendency can be expected, as shown by negative temperature coefficient thermistors. Therefore, temperature dependence can be modeled by the simple thermistor model given by Equation (1).

(1) $$R\left(T\right)=R_{0}\;exp\left[B\;\left(\frac{1}{T}-\frac{1}{T_{0}}\right)\right]$$

where *R(T)* is the resistance at absolute temperature *T*, *R*_0_ is the resistance at the reference temperature *T*_0_ = 298,16 K (25 °C as usually considered for thermistors) and *B* is the material characteristic temperature. A good agreement between experimental data and the thermistor model is achieved in the studied temperature range, as shown in Figure 7, where the average temperature dependence of the three samples is depicted. Let us remember that both LDR devices are made of the same material. As expected, material characteristic temperatures are very similar for both models, around 18,000 K, within the range of the experimental uncertainty. As shown in Figure 7, the sensors have a significant temperature dependence. Therefore, to achieve an accurate dose measurement, two strategies can be followed: (i) To measure the temperature and compensate its influence with Equation (1); (ii) To perform dose measurement in a room with constant temperature, without sensor thermal drift. This last case is quite common in places where radiotherapy treatments are applied.

## 4. Conclusions

In this work, light-dependent resistors have been characterized and tested as dosimetry sensors. It has been experimentally proven that it is feasible to measure the dose rate and the accumulated dose with the selected LDRs, with some limitations such as the use of X-ray beams with dose rates from 50 to 300 cGy/min and considering the electrometer current range in the sensor bias. The current transients and the total charge generated by irradiation were registered per session. From the obtained experimental data, both the dose rate and the accumulated dose can be accurately calculated in a similar way than with ionization chambers. In addition, for the dose rate range analyzed (commonly used in radiotherapy treatments), a linear response was found in all experiments with a minimum correlation factor of R^2^ = 0.986 in the worst case. In the case of the NSL-19M51, the sensitivity varied from (0.24 ± 0.02) to (0.96 ± 0.04) nC/cGy for bias voltages of 30 and 150 V, respectively. The sensitivity of the VT43N2 was (0.34 ± 0.04) and (1.04 ± 0.09) nC/cGy for voltages of 30 and 150 V, respectively. For comparison purposes, the ionization chamber PTW 30010, commonly used in radiotherapy, provides an average sensitivity of 0.2 nC/cGy at 400 V, which is lower than the obtained LDR sensitivities even with lower bias voltage. For temperature correction purposes, the LDR thermal dependence was measured and modeled according to the simple thermistor model. Therefore, commercial LDRs along with a clinical electrometer show a suitable performance at affordable cost to measure the dose rate and the accumulated dose under typical conditions of a radiotherapy treatment.

## Figures and Tables

**Table 1:** LDR sensitivities, a, and correlation factors of the set of analyzed LDRs (covering factor k = 2).

**NSL-19M51**	**(1)**		**(2)**		**(3)**		**Mean**
*I* = a*R* + b	a (nC/cGy)	R^2^	a (nC/cGy)	R^2^	a (nC/cGy)	R^2^	a (nC/cGy)
30 V	0.23 ± 0.02	0.987	0.24 ± 0.03	0.986	0.24 ± 0.02	0.989	0.24 ± 0.02
50 V	0.34 ± 0.03	0.990	0.37 ± 0.04	0.987	0.37 ± 0.04	0.989	0.36 ± 0.03
80 V	0.55 ± 0.05	0.991	0.55 ± 0.06	0.987	0.55 ± 0.06	0.989	0.551 ± 0.004
100 V	0.69 ± 0.07	0.990	0.67 ± 0.08	0.987	0.67 ± 0.07	0.990	0.68 ± 0.02
130 V	0.86 ± 0.09	0.989	0.84 ± 0.10	0.986	0.84 ± 0.08	0.991	0.85 ± 0.03
150 V	0.98 ± 0.10	0.989	0.95 ± 0.11	0.986	0.94 ± 0.09	0.991	0.96 ± 0.04
**VT43N2**	**(1)**		**(2)**		**(3)**		**Mean**
*I* = a*R* + b	a (nC/cGy)	R^2^	a (nC/cGy)	R^2^	a (nC/cGy)	R^2^	a (nC/cGy)
30 V	0.36 ± 0.03	0.994	0.32 ± 0.03	0.992	0.34 ± 0.03	0.993	0.34 ± 0.04
50 V	0.50 ± 0.04	0.993	0.47 ± 0.04	0.991	0.47 ± 0.04	0.991	0.48 ± 0.03
80V	0.68 ± 0.07	0.989	0.63 ± 0.07	0.988	0.62 ± 0.06	0.990	0.65 ± 0.06
100 V	0.81 ± 0.08	0.989	0.74 ± 0.09	0.986	0.74 ± 0.08	0.988	0.76 ± 0.07
130 V	0.97 ± 0.11	0.986	0.89 ± 0.11	0.985	0.92 ± 0.10	0.988	0.92 ± 0.08
150 V	1.08 ± 0.12	0.987	0.99 ± 0.13	0.984	1.04 ± 0.11	0.987	1.04 ± 0.09
